# Small regulatory RNAs as key modulators of antibiotic resistance in pathogenic bacteria

**DOI:** 10.71150/jm.2501027

**Published:** 2025-04-29

**Authors:** Yubin Yang, Hana Hyeon, Minju Joo, Kangseok Lee, Eunkyoung Shin

**Affiliations:** 1 Department of Life Science, Chung-Ang University, Seoul 06974, Republic of Korea; 2 Department of Microbiology, Catholic University of Daegu School of Medicine, Daegu 42472, Republic of Korea

**Keywords:** small regulatory RNAs, antibiotic resistance, post-transcriptional regulation, pathogenic bacteria

## Abstract

The escalating antibiotic resistance crisis poses a significant challenge to global public health, threatening the efficacy of current treatments and driving the emergence of multidrug-resistant pathogens. Among the various factors associated with bacterial antibiotic resistance, small regulatory RNAs (sRNAs) have emerged as pivotal post-transcriptional regulators which orchestrate bacterial adaptation to antibiotic pressure via diverse mechanisms. This review consolidates the current knowledge on sRNA-mediated mechanisms, focusing on drug uptake, drug efflux systems, lipopolysaccharides, cell wall modification, biofilm formation, and mutagenesis. Recent advances in transcriptomics and functional analyses have revealed novel sRNAs and their regulatory networks, expanding our understanding of resistance mechanisms. These findings highlight the potential of targeting sRNA-mediated pathways as an innovative therapeutic strategy to combat antibiotic resistance, and offer promising avenues for managing challenging bacterial infections.

## Introduction

The emergence and global spread of multidrug-resistant (MDR) pathogens is a critical challenge in modern medicine, threatening the efficacy of antibiotics and complicating the management of bacterial infections. Recent global estimates have attributed 4.71 million deaths in 2021 to bacterial antimicrobial resistance (AMR), including 1.14 million deaths directly attributable to bacterial AMR (Collaborators, 2024). This ongoing crisis highlights the critical need for innovative strategies to combat AMR, particularly against MDR pathogens. Among these, ESKAPE pathogens (*Enterococcus faecium*, *Staphylococcus aureus*, *Klebsiella pneumoniae*, *Acinetobacter baumannii*, *Pseudomonas aeruginosa*, and *Enterobacter* species) significantly contribute to AMR and present persistent therapeutic challenges despite the recent introduction of novel antibiotics and β-lactamase inhibitors (Miller and Arias, 2024). These pathogens have adapted to thrive in modern healthcare environments by leveraging diverse mechanisms to acquire resistance determinants and disseminate high-risk clones globally. Advances in next-generation sequencing have enhanced our ability to track the spread and evolution of resistance, while the growing interest in nontraditional therapeutic strategies highlights the urgent need for innovative approaches to combat these pathogens in clinical settings. MDR pathogens not only resist multiple antibiotic classes, but also evade antimicrobial agents and host immune defenses via advanced mechanisms, leading to persistent and life-threatening infections. For example, among Gram-negative bacteria, carbapenem resistance has been linked to more than 1.03 million global deaths by 2021, underscoring the urgent need for effective interventions (Collaborators, 2024). Similarly, methicillin-resistant *Staphylococcus aureus* infections have resulted in a significant increase in global mortality, with associated deaths increasing from 261,000 in 1990 to 550,000 in 2021 and attributable deaths rising from 57,200 to 130,000 during the same period, indicating the growing impact of AMR on global health.

Effectively addressing the challenge of AMR requires a comprehensive understanding of the underlying molecular mechanisms. While protein-based resistance factors, such as efflux pumps and β-lactamases, have been extensively characterized, recent evidence underscores the critical role of small regulatory RNAs (sRNAs) in orchestrating key resistance pathways (Mediati et al., 2021). Notably, sRNAs function independently of translation and exert regulatory effects through base-pairing interactions with target RNAs. These interactions modulate translation, influence messenger RNA (mRNA) stability, and alter transcriptional activity. sRNAs can be categorized into cis- and trans-encoded types. Cis-encoded sRNAs complement their targets and act on RNAs transcribed from the same locus. In contrast, trans-encoded sRNAs, which show partial complementarity to their targets, are transcribed from distinct loci and often depend on chaperone proteins such as Hfq or ProQ for their function (Storz et al., 2011). By regulating processes, such as antibiotic uptake, efflux, biofilm formation, and mutagenesis, sRNAs dynamically respond to environmental stimuli, enabling bacteria to rapidly adapt to antibiotic stress. This remarkable regulatory capacity establishes sRNAs as pivotal contributors to bacterial survival, and highlights their potential as promising targets for novel antimicrobial therapeutic strategies.

This review focuses on the regulatory roles of sRNAs in bacterial antibiotic resistance, specifically, their involvement in controlling mechanisms such as drug uptake, efflux, cell envelope modifications, biofilm formation, and mutagenesis. Additionally, we discuss the potential of targeting sRNA-mediated pathways as a novel therapeutic strategy to combat antibiotic resistance and provide insights into innovative approaches for managing infections caused by clinically significant pathogens.

## Key Mechanisms of Antibiotic Resistance

Antibiotic resistance mechanisms have been extensively studied and well documented, providing a strong basis for understanding how bacteria evade antimicrobial agents. These mechanisms are broadly categorized into the following three main strategies: (i) reducing intracellular antibiotic concentrations, (ii) modifying or protecting antibiotic targets, and (iii) directly inactivating antibiotics (Blair et al., 2015; Darby et al., 2023). Each strategy reflects the remarkable adaptability of bacteria to antibiotic pressure and underscores the challenges in developing effective treatment strategies.

Reducing intracellular antibiotic concentrations is a key mechanism by which bacteria minimize the impact of antibiotics. This can be achieved by decreasing the permeability of bacterial membranes. For instance, the outer membrane of Gram-negative bacteria acts as a protective barrier, blocking the penetration of large hydrophilic compounds, such as glycopeptides, which are unable to reach their targets. Mutations or reduced expression of porin channels like OmpC or OmpF in *Enterobacter* spp. and OmpK35 or OmpK36 in *K. pneumoniae* additionally limit the uptake of β-lactams and carbapenems, further exacerbating resistance by reducing the effectiveness of these antibiotics (Doumith et al., 2009; Jaffe et al., 1982). In addition to limiting antibiotic entry, bacteria actively expel antibiotics from the cells via efflux systems. Efflux pumps such as AcrAB-TolC in *Escherichia coli* and MexAB-OprM in *P. aeruginosa* play pivotal roles in reducing intracellular antibiotic concentrations. These systems can expel multiple classes of antibiotics, including tetracyclines and fluoroquinolones, thereby significantly contributing to MDR. Notably, hospital-associated bacteria, including *S. aureus*, often express high levels of efflux pumps due to regulatory mutations in the promoter region of the efflux pump or its regulatory gene. This heightened expression under intense selective pressure in hospital environments highlights the critical role of efflux systems in both intrinsic bacterial survival and development of clinically significant AMR (Henderson et al., 2021a; Kosmidis et al., 2012).

Bacteria can also evade the action of antibiotics by altering the structure or accessibility of drug targets through various mechanisms. These include mutations in genes encoding the target, enzymatic modifications of the antibiotic-binding site, or protection of the target, which collectively reduce the antibiotic-binding affinity while preserving target functionality. For example, point mutations in the quinolone resistance-determining regions of DNA gyrase and topoisomerase IV confer resistance to fluoroquinolones in *S. aureus* (Bush et al., 2020; Hooper and Jacoby, 2015). Similarly, enzymatic modification of the antibiotic-binding site is exemplified by *erm* genes, which encode ribosomal methyltransferases. These enzymes methylate the active site of the ribosome and confer resistance to macrolides by preventing their binding (Svetlov et al., 2021). Linezolid resistance in *S. aureus* occurs via various mechanisms. These include mutations in domain V of 23S rRNA (e.g., G2576), methylation by *cfr* (chloramphenicol-florfenicol resistance)-encoded methylase, and the action of ribosomal protection proteins such as those encoded by *optrA* and *poxtA* (Yang et al., 2024). These mechanisms disrupt the binding of linezolid to the peptidyl transferase center by altering its structure or accessibility, ultimately reducing the efficacy of the drug and leading to treatment challenges in resistant *S. aureus* strains.

Bacteria can also acquire resistance through enzymatic modification or degradation of antibiotics, which represents a distinct and clinically significant strategy. This approach neutralizes antibiotic efficacy by directly targeting the drug itself rather than modifying the bacterial target, allowing essential cellular functions to remain intact while rendering the antibiotic ineffective (Schaenzer and Wright, 2020). β-Lactamases, including extended-spectrum β-lactamases and carbapenemases, hydrolyze β-lactam antibiotics, rendering them ineffective (Tooke et al., 2019). Dissemination of the enzyme-coding genes, such as *bla*_CTX-M_ and *bla*_NDM-1_, through plasmids has accelerated the global spread of highly resistant pathogens, such as *K. pneumoniae* and *A. baumannii*, leading to significant clinical challenges (Bradford, 2001; Chen et al., 2012). Similarly, aminoglycoside-modifying enzymes, such as nucleotidyltransferases, phosphotransferases, and acetyltransferases, add chemical groups (e.g., acetyl and phosphate) to aminoglycosides, thus disrupting their interaction with the bacterial ribosome (Ramirez and Tolmasky, 2010). This enzymatic alteration significantly diminishes the antibacterial activity of aminoglycosides and contributes to widespread resistance observed in clinically relevant pathogens.

## The Role of sRNAs in Bacterial Gene Regulation

sRNAs are versatile noncoding molecules which play essential roles in regulating cellular processes across all domains of life. In bacteria, sRNAs typically range in size from 50 to 500 nucleotides and regulate gene expression by interacting with DNA, proteins, other RNA molecules, and metabolites (Storz et al., 2011). Unlike protein-coding genes, sRNAs do not encode proteins, but modulate gene expression post-transcriptionally, primarily through interactions with mRNAs. This ability to influence gene expression in response to environmental changes such as antibiotic exposure, nutrient availability, and stress enables bacteria to adapt rapidly, making sRNAs critical for bacterial survival, virulence, and persistence (Chabelskaya et al., 2010; Romilly et al., 2014; Westermann, 2018).

**Fig. 1.**
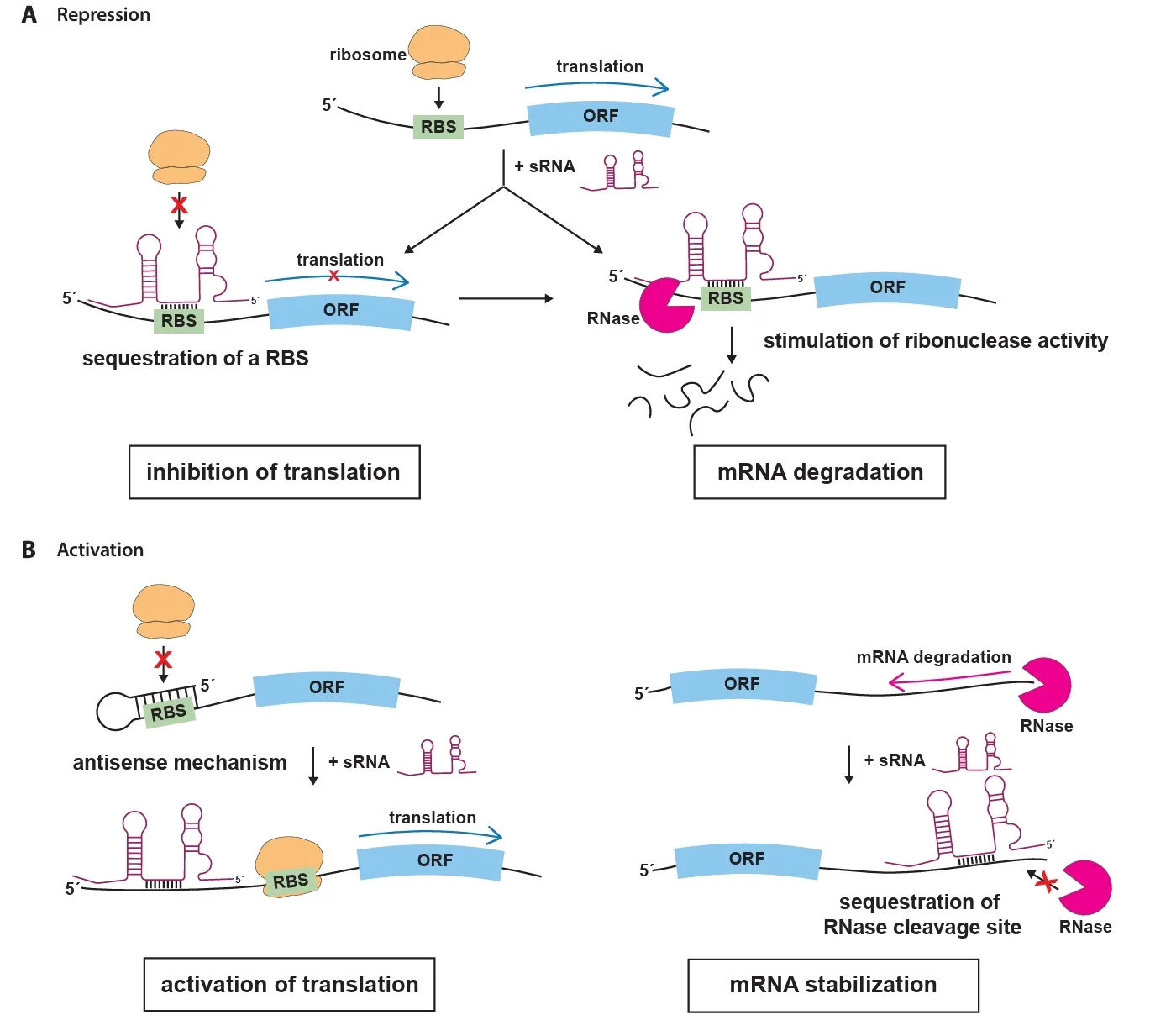
Mechanisms of sRNA-mediated regulation in bacteria. (A) Trans-acting sRNAs can bind to the 5′ untranslated region (UTR) of target mRNAs, masking the ribosome binding site and preventing ribosome access, which leads to translational inhibition. sRNAs can also pair with regions upstream of the start codon (AUG) of target mRNAs, forming sRNA-mRNA duplexes that are recognized and degraded by ribonucelase, resulting in effective and irreversible repression of translation. (B) In a positive regulatory role, sRNAs may disrupt inhibitory secondary structures within the mRNA that conceal the RBS. By exposing the RBS, the binding of ribosomes is facilitated, thereby promoting translation. sRNAs can bind to RNase cleavage sites on target mRNAs, preventing degradation by blocking RNase access. This stabilization indirectly increases the concentration of stable mRNAs, leading to enhanced protein production. Abbreviations: ORF, open reading frame; RBS, ribosome binding sites; RNase, ribonuclease.

The most extensively studied group of sRNAs is the trans-encoded sRNAs, which regulate gene expression through short, often imperfect, base-pairing interactions with their target mRNAs (Waters and Storz, 2009). These interactions typically occur in the 5′ untranslated region (UTR) of the target mRNA, where sRNAs can prevent translation initiation by blocking the ribosome binding site (RBS), effectively preventing ribosome binding and translation (Fig. 1A). Moreover, the loss of ribosome protection caused by sRNA binding makes the mRNA vulnerable to RNase E-mediated cleavage, followed by exonucleolytic decay (Dreyfus, 2009; Massé et al., 2003). In some cases, sRNAs can enhance mRNA translation by inducing structural rearrangements or stabilize mRNAs by blocking nuclease cleavage sites to prevent degradation (Fig. 1B). In some cases, sRNAs can enhance mRNA translation by inducing structural rearrangements or stabilize mRNAs by blocking nuclease cleavage sites to prevent degradation. For example, they can disrupt inhibitory secondary structures within the mRNA that occlude the RBS, allowing ribosomes to access the RBS and initiate translation. Alternatively, sRNAs can bind near RNase cleavage sites, preventing degradation by nucleases and ensuring the stability of the target mRNA (Bossi and Figueroa-Bossi, 2016; Papenfort and Vanderpool, 2015).

Many of these sRNAs regulate multiple mRNA targets and often require the assistance of RNA chaperones, such as Hfq, ProQ, and CspA, which stabilize the sRNA and facilitate its interaction with target mRNAs (Quendera et al., 2020; Smirnov et al., 2017; Storz et al., 2011). For example, Hfq is required for the activity and stability of many trans-encoded sRNAs, facilitating efficient interactions between sRNAs and their mRNA targets in Gram-negative bacteria. Hfq acts as a platform for sRNAs and mRNAs to sample potential complementarity, thereby increasing their local concentrations. These base-pairing sRNAs serve as key posttranscriptional regulators which modulate mRNA decay and translation by promoting or inhibiting these processes. In addition, some sRNAs regulate gene expression by binding to regulatory proteins, often through sequestration, which modulates the activity of these proteins (Argaman et al., 2001). For example, **SgrS** in *E. coli* sequesters CsrA, a translational repressor, preventing it from inhibiting the translation of genes involved in biofilm formation and virulence under stressful conditions (Maki et al., 2008).

Recent advancements in high-throughput RNA profiling technologies, such as ‘RNA interaction by ligation and sequencing (RIL-seq)’ and ‘crosslinking, ligation, and sequencing of hybrids (CLASH)’, have significantly enhanced our understanding of sRNA-mediated regulation (Helwak et al., 2013; Kudla et al., 2011; Melamed et al., 2016, 2018; Waters et al., 2017). These methods have revealed the complexity of the sRNA-mRNA interactome, demonstrating how sRNAs regulate gene expression by interacting with various genomic regions, including antisense to coding regions, 3′ and 5′ UTRs, and even coding regions. Additionally, techniques such as UV crosslinking and RNA analysis using cDNA (CRAC-seq), nanopore-based sequencing for sRNAs (SR-Cat-Seq), and gradient profiling by sequencing (Grad-seq) have enabled the detailed tracking of sRNA interactions, further underscoring their critical role in bacterial adaptation to stress and environmental changes (Hor et al., 2018, 2020; Maguire and Guan, 2022; Sy et al., 2018; Tree et al., 2014).

sRNAs are critical for modulating various cellular processes, including antibiotic susceptibility, by regulating mRNAs involved in drug uptake, drug efflux, cell envelope modifications, biofilm formation, and DNA mutagenesis (Table 1). As key post-transcriptional regulators, sRNAs coordinate the expression of multiple genes, enabling bacteria to rapidly adapt to environmental stressors, such as antibiotic exposure. This adaptability ensures bacterial survival and positions sRNAs as promising targets for therapeutic strategies against MDR pathogens. Ongoing research on sRNA-mediated regulation offers great potential for advancing our understanding of bacterial resistance mechanisms and developing novel antimicrobial therapies. Although the detailed mechanisms of sRNA regulation have been reported, here we focus on their clinical relevance, particularly in relation to MDR pathogens.

## Mechanisms of sRNA-mediated Antibiotic Resistance

### sRNA-mediated control of drug uptake

**Fig. 2.**
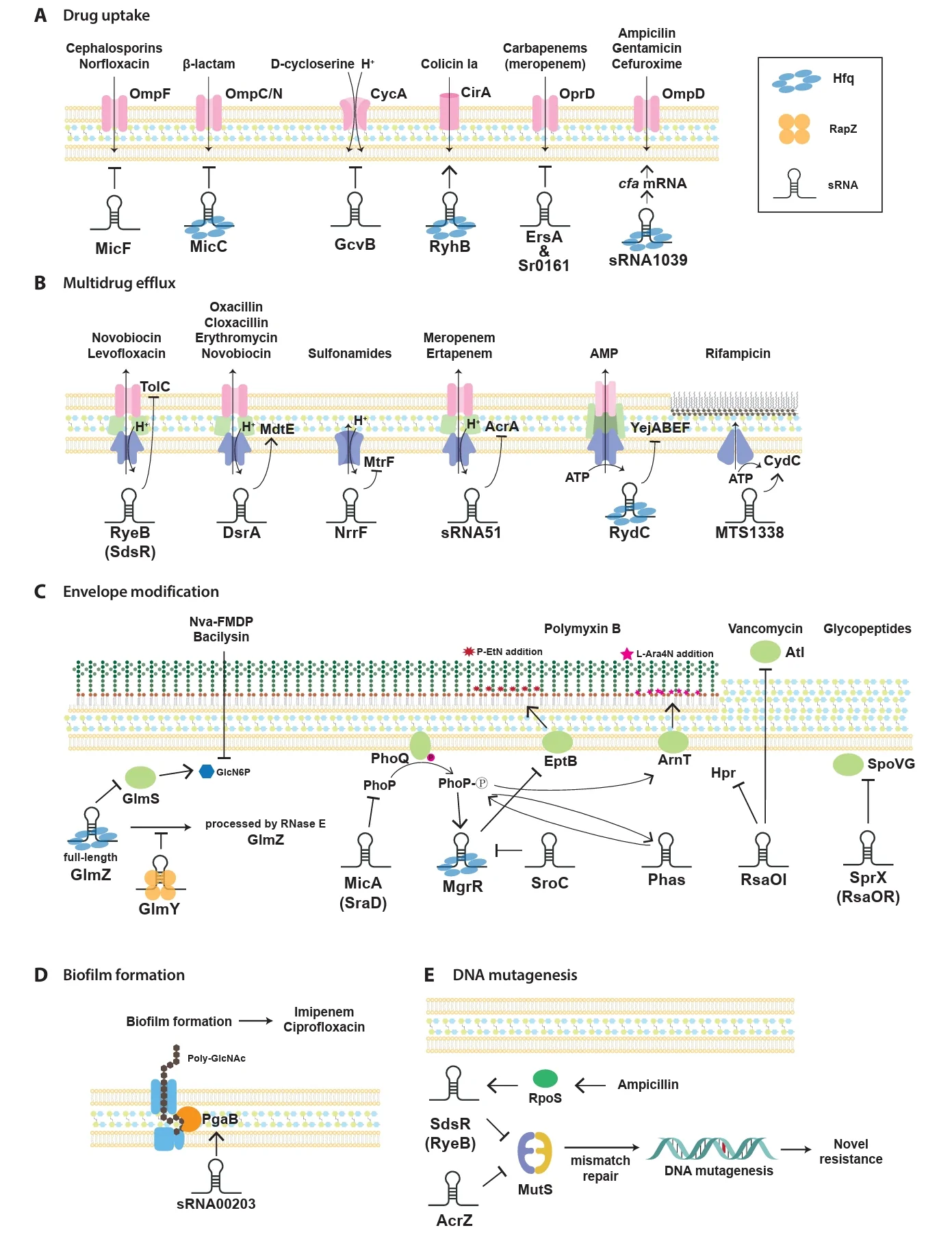
Regulation mechanisms of antibiotic-resistance by sRNA in pathogenic bacteria. Mechanisms influenced by representative sRNA-mediated antibiotic response and resistance highlighted in this study are categorized into five key sections: drug uptake (A), multidrug efflux (B), envelope modification (C), biofilm formation (D), and DNA mutagenesis (E). Antibiotics regulated by sRNA mechanisms and their prominent sRNA targets are highlighted. Arrows represent the upregulation of target gene expression driven by sRNAs, while flat-ended lines indicate sRNA-mediated downregulation of target gene expression. Abbreviations: AMP, antimicrobial peptide.

sRNAs have been progressively identified as key regulators of antibiotic resistance, which act through various mechanisms to modulate bacterial adaptability. First, sRNAs significantly influence bacterial antibiotic resistance by regulating drug uptake through the modulation of membrane permeability (Fig. 2A). This process typically involves the regulation of porins, which are channels facilitating antibiotic entry into bacterial cells. By altering porin expression, sRNAs reduce the influx of antibiotics, thereby lowering their intracellular concentrations and conferring resistance. In *E. coli*, sRNAs modulate the expression of outer membrane porins and transporters, which alters bacterial susceptibility to specific antibiotics. For instance, the sRNA **MicF** suppresses the translation of *ompF*, a major outer membrane porin responsible for antibiotic entry (Nikaido, 1989). Reduced OmpF levels limit the influx of antibiotics, such as cephalosporins, minocycline, and norfloxacin, thereby decreasing bacterial susceptibility. Conversely, *micF* deletion restores OmpF expression, leading to increased sensitivity to these antibiotics, except for minocycline (Kim et al., 2015). Similarly, the conserved Hfq-associated sRNA **MicC** controls the OmpC/N levels in response to β-lactam antibiotics (Chen et al., 2004; Dam et al., 2017). In addition, MicF represses *obgE* under oxidative stress and *seqA* under nutrient-rich conditions, highlighting its broad significance in the management of diverse stress responses (Stibelman et al., 2024).

Furthermore, the sRNA **GcvB** represses *cycA*, which encodes a transporter for serine and D-cycloserine uptake (Pulvermacher et al., 2009). Loss of GcvB leads to elevated *cycA* mRNA levels, resulting in increased D-cycloserine uptake and enhanced bacterial susceptibility. In addition to repressing its targets directly, GcvB also enhances RNase E-mediated cleavage at specific sites, leading to transcript destabilization and efficient downregulation of its target genes, highlighting a cooperative mechanism between GcvB and RNase E in modulating bacterial gene expression (Vigoda et al., 2024).

In *E. coli*, **RyhB** regulates antibiotic persistence under iron-limited conditions by repressing metabolism and modulating the translation of the iron-siderophore receptor *cirA*. By displacing the RNA chaperone Hfq, RyhB stabilizes *cirA* mRNA, which promotes colicin Ia uptake and influences bacterial sensitivity to the toxin, highlighting the dual role of sRNA in persistence and susceptibility (Salvail et al., 2013). In uropathogenic *E. coli*, **RyhB** mediates the persistence of various antibiotics such as levofloxacin and cefotaxime by reducing cellular metabolism (Porcheron et al., 2014; Zhang et al., 2018). As a key regulator of iron homeostasis, RyhB promotes phenotypic resistance to gentamicin, an aminoglycoside which targets ribosomes during iron starvation (Chareyre et al., 2019). In *P. aeruginosa*, the OprD porin, which enables the uptake of carbapenem antibiotics, is post-transcriptionally controlled by two sRNAs, **Sr0161** and **ErsA**. These sRNAs suppress *oprD* mRNA levels and OprD protein expression, thereby reducing the susceptibility to carbapenem antibiotics, including meropenem (Zhang et al., 2017). Recent studies have documented such cases involving various clinically significant pathogens. For example, Hfq-dependent **sRNA1039** regulates the resistance of *Shigella sonnei* to gentamicin, ampicillin, and cefuroxime, and bacterial growth. The deletion of sRNA1039 accelerates the degradation of its target *cfa* mRNA, reducing its expression and indirectly downregulating the pore protein gene *ompD*, thereby increasing bacterial sensitivity to these antibiotics. In this study, they identified an additional sRNA, **sRNA1600**, which is presumably involved in regulating *S. sonnei* resistance to cefuroxime and modulating virulence by influencing persister cell formation (Du et al., 2024). *Burkholderia cenocepacia*, a Gram-negative bacterium which causes infections in immunocompromised individuals and people with cystic fibrosis, uses the sRNA **NcS25**. This sRNA controls the outer membrane porin BCAL3473, which is responsible for transporting biological amines, emphasizing the importance of sgRNAs in regulating membrane transport and facilitating antibiotic uptake (Sass and Coenye, 2023).

### Regulation of efflux pumps

Multidrug efflux pumps, which actively expel antibiotics and toxic compounds to reduce intracellular drug accumulation, are major contributors to bacterial drug resistance (Fig. 2B). Efflux pumps in bacteria are classified into the following five distinct families based on their structural composition, number of transmembrane domains, energy sources, and substrate specificity: resistance-nodulation-division (RND) family, ATP-binding cassette (ABC) superfamily, multidrug and toxic compound extrusion (MATE) family, major facilitator superfamily (MFS), and small multidrug resistance (SMR) family (a subset of the broader drug/metabolite transporter superfamily) (Li and Nikaido, 2009; Piddock, 2006). Notably, while RND efflux pumps are exclusively found in Gram-negative bacteria, the other four families (ABC, MATE, MFS, and SMR) are broadly distributed across both Gram-negative and Gram-positive bacterial species (Handzlik et al., 2013). Notably, efflux pump regulation, particularly through sRNA-mediated mechanisms, represents another critical layer of control in the development of antibiotic resistance (Nikaido, 1996; Zgurskaya and Nikaido, 2000).

The sRNA **RyeB** (also known as **SdsR**), which is abundantly expressed during the stationary phase, suppresses *tolC*, a key component of the AcrAB-TolC efflux system responsible for expelling lipophilic antibiotics in *E. coli* and *Salmonella enterica* serovar Typhimurium (Fröhlich et al., 2016; Kim et al., 2015; Parker and Gottesman, 2016; Vogel et al., 2003). Overexpression of RyeB reduces TolC levels and increases susceptibility to novobiocin and quinolone antibiotics such as levofloxacin, whereas its absence allows robust *tolC* expression, sustaining active drug efflux and resistance (Kim et al., 2015; Parker and Gottesman, 2016).

**DsrA** (downstream of *rcsA*), which was initially identified as a regulator of capsular polysaccharide synthesis and pathogenicity factors, enhances multidrug resistance in *E. coli* by upregulating the MdtEF efflux pump via the RpoS pathway. Using a random shotgun cloning approach, it was revealed that DsrA significantly increases *mdtE* expression and decreases susceptibility to antibiotics such as oxacillin, cloxacillin, erythromycin, and novobiocin (Nishino et al., 2011). In *Neisseria* species, the sRNA **NrrF** (neisserial regulatory RNA responsive to iron [Fe]) serves as a key transcriptional regulator of efflux pump synthesis by modulating the *sdh* operon, and overexpression studies have demonstrated its ability to suppress antibiotic-induced efflux pump expression (Jackson et al., 2013; Mellin et al., 2007). NrrF modulates the mRNA levels of *mtrF*, a tightly regulated accessory protein of the MtrCDE efflux pump, which contributes to bacterial resistance by extruding antimetabolites, specifically sulphonamides (Su et al., 2015). In the nosocomial pathogen *A. baumannii*, **AbsR25** (*Acinetobacter baumannii* small RNA25) negatively regulates the expression of *abaF* gene, which encodes a putative MFS transporter involved in efflux pump activity. Here, increasing EtBr concentrations suppresses AbsR25 mRNA expression, leading to upregulation of the efflux pump and subsequent MDR (Sharma et al., 2014, 2017).

Recent studies have advanced our understanding of the sRNA-mediated regulation of bacterial antibiotic resistance, with a particular focus on the control of multidrug efflux pumps in clinically significant pathogens and antibiotic-resistant strains. **sRNA51** is a key regulator of carbapenem-resistant *Klebsiella pneumoniae* strains which produce KPC-2, a class A carbapenemase, responsible for carbapenem resistance. It downregulates *acrA*, leading to reduced efflux pump activity and the restoration of susceptibility to meropenem and ertapenem (Bai et al., 2024). In *Mycobacterium tuberculosis*, rifampicin treatment strongly upregulates the sRNA MTS1338 by more than four-fold. High intracellular **MTS1338** levels enhance bacterial growth under rifampicin exposure by stabilizing the mRNA of the efflux protein CydC through direct binding, thereby protecting it from degradation. This mechanism reduces intracellular antibiotic accumulation in *M. tuberculosis*, highlighting its role in the adaptation to antibiotic stress (Singh and Dutta, 2024).

Antimicrobial peptides (AMPs) have emerged as promising alternatives to traditional antibiotics owing to their low rates of resistance development and ability to primarily target the bacterial membrane (Ryu et al., 2021). However, bacteria can regulate AMP resistance through sRNA-mediated modulation of genes involved in transporter synthesis, which plays a critical role in efflux-mediated defense mechanisms. For instance, **RydC**, a pseudoknot-folding sRNA which binds Hfq, regulates the ABC transport system by promoting the degradation of its mRNA target, *yejABEF*, in *E. coli*, *Salmonella*, and *Shigella* (Antal et al., 2005). The *yejABEF* operon, which encodes an ABC transporter, facilitates AMP resistance by driving active AMP efflux in *Salmonella* and *Brucella melitensis* (Cao et al., 2019; Eswarappa et al., 2008; Wang et al., 2016). Collectively, these findings highlight the pivotal role of sRNAs in regulating efflux systems and facilitating bacterial adaptation to antibiotic stress.

### sRNA-mediated regulation of cell envelope modification

The Gram-negative cell envelope consists of an inner membrane (IM), a periplasmic space containing peptidoglycan (PG), and an outer membrane (OM) (Beveridge, 1999). Lipopolysaccharides (LPS), an essential component of the OM, play a crucial role as both a structural barrier and a key virulence factor, ensuring OM integrity in most Gram-negative bacteria (Zhang et al., 2013). LPS biosynthesis and modification are precisely regulated through a complex network of transcription factors and two-component systems, such as PhoP/PhoQ and PmrA/PmrB, which respond to environmental signals to modulate LPS composition and structure. These regulatory layers ensure that the LPS structure and composition are dynamically adjusted in response to environmental stresses, host immune pressures, and antimicrobial treatments (Rosenfeld and Shai, 2006). At the post-transcriptional level, sRNAs fine-tune LPS biosynthesis and modifications by directly targeting mRNAs. These interactions regulate key processes, such as precursor availability, enzymatic modifications of LPS, and the incorporation of lipid A modifications (Needham and Trent, 2013; Raetz and Whitfield, 2002). By coordinating these pathways, sRNAs maintain structural integrity of the OM and also contribute to bacterial survival under stress and resistance to AMPs, such as polymyxins (Fig. 2C).

The **GlmY/GlmZ** network, is a well-established sRNA regulatory system which plays a pivotal role in modulating LPS biosynthesis by controlling the expression of *glmS*, a cell-wall biosynthesis enzyme critical for the production of glucosamine-6-phosphate (GlcN6P), a key precursor for LPS assembly (Kalamorz et al., 2007; Reichenbach et al., 2008; Urban and Vogel, 2008). GlmZ directly promotes *glmS* translation by base pairing with its mRNA, thereby alleviating the formation of inhibitory secondary structures at the ribosome-binding site and facilitating translation initiation. This regulatory mechanism depends on the unprocessed form of GlmZ, which accumulates under low GlcN6P conditions with upstream stabilization by the sRNA GlmY (Urban and Vogel, 2008). GlmY stabilizes GlmZ by interfering with the interaction between GlmZ and the adaptor protein RapZ, thereby preventing the RNase E-mediated degradation of GlmZ (Durica-Mitic et al., 2020; Reichenbach et al., 2008). This hierarchical regulation ensures sufficient GlcN6P production, particularly under nutrient-limiting conditions, underscoring the critical role of sRNAs in coordinating the metabolic requirements for cell envelope assembly and bacterial survival.

Another well-characterized sRNA involved in LPS regulation is **MicA** (also known as **SraD**), which modulates multiple genes critical for LPS modification in *E. coli*. MicA represses the PhoP/Q two-component system, thereby reducing the addition of phosphoethanolamine (P-EtN) to lipid A, a modification which influences bacterial resistance to cationic AMPs such as polymyxin (Coornaert et al., 2010; Gogol et al., 2011). These coordinated regulatory actions allow MicA to adjust the LPS composition and contribute to bacterial adaptation under stress.

**MgrR** is a key Hfq-dependent sRNA, whose expression is activated by the PhoQ/PhoP two-component system under low Mg^2+^ conditions. It represses *eptB*, a gene encoding a protein that incorporates P-EtN into the second Kdo residue of LPS, thus maintaining the anionic charge of LPS, which is critical for the susceptibility to polymyxins. Notably, *mgrR* deletion leads to a 10-fold increase in resistance to polymyxin B, highlighting its pivotal role in modulating LPS structure and antibiotic sensitivity in response to environmental Mg^2+^ levels (Klein et al., 2011; Moon and Gottesman, 2009). Furthermore, **SroC**, an sRNA sponge, sequesters MgrR and indirectly regulates LPS modification by regulating the repression of *eptB*. Deletion of SroC increases free MgrR levels, which represses *eptB* and enhances susceptibility to polymyxin B by maintaining the anionic charge of LPS in *S. Typhimurium* (Acuna et al., 2016). In contrast, recent research on carbapenem-resistant *Klebsiella pneumoniae* has identified **PhaS** as a key enhancer of polymyxin B heteroresistance by regulating the PhoP/Q system. PhaS upregulates *phoP*, which subsequently activates *pmrD* and *arnT*, driving the L-Ara4N modification of lipid A to reduce susceptibility to polymyxin B. Notably, a positive feedback loop has been revealed, where PhoP further amplifies PhaS expression under antibiotic stress, highlighting a novel resistance mechanism (Zhao et al., 2024). These findings highlight the pivotal role of sRNAs as master regulators of LPS biosynthesis and modification, which regulate critical pathways to ensure bacterial survival and adaptation. By integrating signals from diverse environmental cues, sRNAs coordinate complex regulatory networks which not only maintain OM integrity but also enhance bacterial resilience against host immune responses and antimicrobial agents. Elucidating the precise molecular mechanisms of sRNA-mediated regulation offers promising opportunities to uncover novel therapeutic targets and pave the way for innovative strategies to treat infections caused by antibiotic-resistant bacterial pathogens.

Recent research has highlighted the role of the vancomycin-responsive sRNA **RsaOI** as a significant regulator of vancomycin tolerance in *S. aureus* (Wu et al., 2024). RsaOI exerts its regulatory effects through the post-transcriptional repression of key target mRNAs, including HPr, which governs carbon metabolism, and Atl, a cell wall autolysin involved in cell wall dynamics. Using RNase III-CLASH and self-organizing maps, RsaOI suppresses translation and facilitates mRNA degradation, revealing a novel pathway which associates sRNA-mediated regulation to antibiotic tolerance mechanisms in *S. aureus*. Furthermore, the sRNA **SprX** (also known as **RsaOR**) in *S. aureus* regulates glycopeptide resistance by targeting the *yabJ-spoVG* operon. SprX inhibits SpoVG, a key transcription factor involved in glycopeptide resistance, through antisense interactions at internal translation initiation sites, without altering the overall mRNA levels or translation of YabJ, the upstream gene. The CU-rich region of SprX plays a critical role in this process. In addition to SpoVG, SprX may influence additional pathways linked to glycopeptide resistance, suggesting its broad involvement in sRNA-mediated networks in Gram-positive bacteria. Nevertheless, studies on sRNA-regulated antibiotic resistance mechanisms in Gram-positive bacteria, particularly those involving cell envelope modifications, remain limited, highlighting the importance of such research in broadening our knowledge of the glycopeptide resistance mechanisms including vancomycin, in *S. aureus*.

### sRNA-mediated regulation of biofilm formation

Biofilm formation is a crucial survival strategy for bacteria which allows them to thrive in hostile environments by adopting a surface-attached, matrix-encased structure (Costerton et al., 1999). This structure limits antimicrobial penetration, alters the microenvironment, and supports the survival of dormant cells. Furthermore, biofilms enhance resistance through multidrug efflux pumps, extracellular DNA, and stress response pathways, ultimately contributing to chronic infections which are difficult to treat with conventional antibiotics. Among the various regulatory mechanisms that sustain biofilm-associated resistance, sRNAs play an important role in regulating key processes, such as surface attachment, quorum sensing, motility, and extracellular matrix production (Chambers and Sauer, 2013). These pathways enable bacteria to dynamically adapt to environmental challenges and persist under antibiotic pressure. As biofilms are major contributors to antibiotic resistance, understanding sRNA-mediated regulation provides valuable insights into the mechanisms that sustain bacterial persistence and offers new opportunities for targeted therapeutic strategies (Fig. 2D).

In *E. coli* and *Salmonella*, the transcriptional regulator, CsgD, determines biofilm formation by facilitating curli fimbriae production and secretion, while repressing flagellar biosynthesis genes (Ogasawara et al., 2011; Römling et al., 1998). In *E. coli*, numerous Hfq-dependent sRNAs (GcvB, OmrA, OmrB, McaS, and RprA) modulate *csgD* mRNA levels by base-pairing with its 5′ UTR, blocking ribosome binding and interfering with translational initiation (Chambers and Sauer, 2013). These sRNAs act as repressors under various growth conditions and regulate the biofilm-associated processes. In *S. Typhimurium*, CsgD regulates the transition between planktonic and biofilm growth, contributing to the rdar morphotype through curli fimbriae and cellulose production. *Salmonella* employs distinct sRNAs such as SdsR and ArcZ to regulate CsgD expression. **SdsR** is critical for maintaining steady-state levels of *csgD*, whereas **ArcZ** represses *fliC*, a flagellar machinery gene and regulates RpoS, a sigma factor involved in stress adaptation (Mandin and Gottesman, 2010; Monteiro et al., 2012). Additional sRNAs, including **RprA** and **DsrA**, activate RpoS translation under specific stress conditions, thus enhancing biofilm formation and persistence (Majdalani et al., 2001).

In *P. aeruginosa*, the RsmA protein, a homolog of CsrA, is antagonized by the sRNAs **RsmY** and **RsmZ**, which operate under the control of the GacA-GacS two-component system (Mikkelsen et al., 2011). These sRNAs promote biofilm formation by suppressing motility and virulence factors such as the type III secretion system. However, elevated RsmY and RsmZ levels, while enhancing the initial attachment, can hinder biofilm maturation, indicating the importance of tight regulation. **PrrF** sRNAs repress iron-containing proteins during iron starvation by specifically targeting enzymes, such as succinate dehydrogenase and aconitase, which are involved in the tricarboxylic acid cycle. Additionally, PrrF promotes production of the *Pseudomonas* quinolone signal, a quorum-sensing molecule which activates virulence genes (Reinhart et al., 2015). Recent studies have also identified **PA0730.1**, an sRNA in *P. aeruginosa*, which regulates growth rate, motility, and biofilm formation by modulating the expressions of *mucA* and *rpoS* at both the transcriptional and translational levels. These findings underscore the intricate regulatory networks involved in bacterial pathogenesis and environmental adaptation, and highlight the multifaceted roles of sRNA-mediated pathways in biofilm regulation (Kar et al., 2024). In *A. baumannii*, the novel sRNA **sRNA00203** regulates biofilm formation by modulating expression of the *pgaB* gene, which is involved in biofilm matrix synthesis. Additionally, sRNA influence the expression of genes associated with LPS biosynthesis (novel00626), quorum sensing (*secA* and CRP transcriptional regulator), and efflux pump production (novel00738), thereby altering resistance to biofilm-specific antibiotics, such as imipenem and ciprofloxacin (Shenkutie et al., 2023).

Recent research has also highlighted the importance of sRNAs in Gram-positive species. In *S. aureus*, **RsaA** enhances biofilm formation by repressing *mgrA*, a global regulator of biofilm-disfavoring genes (Tomasini et al., 2017). **RNAIII** promotes biofilm development by stimulating the Agr quorum-sensing pathway, which coordinates bacterial adhesion and virulent gene expression (Gupta et al., 2015; Tomasini et al., 2017). In *Staphylococcus epidermidis*, **RsaE** sRNA regulates polysaccharide intercellular adhesin-mediated biofilm formation by controlling *icaADBC* operon expression and extracellular DNA release (Schoenfelder et al., 2019). In *Streptococcus mutans*, **FasX** inhibits biofilm formation by repressing collagen-binding pili, whereas **sRNA0426** positively regulates exopolysaccharide biosynthesis, a critical component of biofilm structure. In *Enterococcus faecalis*, the antisense RNA **ASwalR** reduces biofilm formation by post-transcriptionally regulating the WalR response regulator and decreasing exopolysaccharide synthesis and pathogenicity in vivo (Wu et al., 2021).

Building upon other key regulatory networks such as RpoS and CsgD, the Csr system provides an additional layer of control for biofilm formation and bacterial adaptation. CsrA, a global RNA-binding protein, represses genes required for biofilm formation, including those involved in exopolysaccharide synthesis. This repression is alleviated by sRNAs **CsrB** and **CsrC**, which sequester CsrA through multiple GGA recognition sites (Weilbacher et al., 2003). The BarA-UvrY two-component system regulates the transcription of CsrB and CsrC in response to environmental signals such as nutrient limitation or stress, linking external conditions to biofilm adaptation. Additionally, other sRNAs, such as **McaS** and **Spot42**, interact with CsrA to modulate biofilm-related processes, demonstrating the versatility of this regulatory network (Rojano-Nisimura et al., 2023). Unlike other systems, such as RsmA or PrrF, the Csr system uniquely integrates carbon metabolism with biofilm formation, highlighting its distinct yet complementary role in bacterial adaptation.

Collectively, these findings underscore the central role of sRNAs in regulating biofilm formation and bacterial adaptation across diverse species. In Gram-negative bacteria such as *E. coli*, *Salmonella*, and *P. aeruginosa*, sRNAs regulate biofilm-related processes by modulating regulators, such as CsgD, RsmA, RpoS, and CsrA. In Gram-positive bacteria, such as *S. aureus* and *E. faecalis*, sRNAs orchestrate unique pathways to control adhesion, matrix production, and stress responses. Notably, the Csr system highlights the versatility of sRNAs in integrating biofilm regulation with carbon metabolism, thereby adding an additional layer of complexity to bacterial adaptation. Elucidating these mechanisms can potentially reveal novel therapeutic targets for combating biofilm-associated infections and antibiotic resistance.

### sRNAs in DNA mutagenesis

sRNAs play a significant role in influencing mutation rates, thereby enabling bacterial adaptation to antibiotic stress (Fig. 2E). Sub-inhibitory antibiotic concentrations exert survival pressure and activate adaptive mechanisms which drive genetic diversity and foster the development of antibiotic resistance (Gutierrez et al., 2013). For instance, sub-inhibitory concentrations of β-lactam antibiotics, such as ampicillin, trigger mutagenesis by upregulating the stress-response regulator RpoS, which downregulates mismatch-repair activity (Mathieu et al., 2016). The expression of *rpoS* mRNA is tightly regulated at the post-transcriptional level via base-pairing interactions with multiple sRNAs, including RprA, which can either enhance or inhibit its stability and translation (Sedlyarova et al., 2016; Zhang et al., 2018). This regulation increases RpoS activity, thus inducing stress tolerance genes and upregulating error-prone DNA polymerase IV (*dinB*). Polymerase IV introduces mutations into the bacterial genome, enhancing genetic diversity and facilitating the emergence of resistance mechanisms (Gutierrez et al., 2013; Mathieu et al., 2016).

In addition, Gutierrez et al. (2013) demonstrated that RpoS regulates expression of the sRNA SdsR, which is involved in bacterial mismatch repair and mutagenesis under antibiotic stress. **SdsR**, identified as the first sRNA associated with antibiotic stress responses, regulates mismatch repair by targeting *mutS*, a key component of the mismatch repair system, and also plays roles in multiple regulatory pathways with different target genes. By base pairing with *mutS* mRNA, SdsR impedes translation, diminishes mismatch-repair efficiency, and promotes mutagenesis. This allows for the accumulation of mutations which can enhance genetic diversity and potentially confer antibiotic resistance in *E. coli*, *Vibrio cholerae*, and *P. aeruginosa*. **ArcZ** sRNA, along with Hfq, represses the expression of *mutS* mRNA, which encodes an essential component of the mismatch repair system and contributes to regulating stress-induced mutagenesis in *E. coli* (Chen and Gottesman, 2017). The upregulation of error-prone polymerase IV by RpoS, combined with the suppression of *mutS* by SdsR or ArcZ, represents a synergistic mechanism which enhances genomic variability under stressful conditions. Recent studies have revealed additional functions of SdsRs in regulating virulence pathways beyond its established role in promoting genetic diversity and antibiotic resistance. In *S. Typhimurium*, SdsR also plays a significant role in stabilizing mRNAs such as *hilD*, a key virulence regulator, thereby contributing to the precise modulation of virulence gene expression in response to host environmental conditions (Abdulla et al., 2023). The dual function of SdsRs, which governs both antibiotic resistance and virulence, highlights the diverse and essential roles of sRNAs in bacterial adaptation and survival. Together, these findings underscore the central importance of sRNAs in orchestrating complex regulatory networks which allow bacteria to respond efficiently to environmental stresses, host immune defenses, and antibiotic pressures.

## sRNAs as Novel Targets for Antimicrobial Therapy

sRNAs have emerged not only as key regulators of bacterial gene expression but also as promising targets for novel antimicrobial therapies. Considering their central roles in essential bacterial processes such as virulence, stress response, and antibiotic resistance, targeting sRNAs offers a unique opportunity to disrupt bacterial adaptation and survival mechanisms. Recent studies have demonstrated the feasibility of designing therapeutic interventions that either inhibit sRNA function or harness their regulatory potential for antimicrobial purposes (Chan et al., 2017; Dersch et al., 2017; Parmeciano Di Noto et al., 2019). Antisense oligonucleotides (ASOs) can hybridize with complementary sRNA sequences, effectively blocking their interaction with target mRNAs and altering gene expression (Davidson and McCray, 2011; Kole et al., 2012). This approach can disrupt their regulatory functions and restore bacterial susceptibility to antibiotics. For example, ASOs targeting MicF in *E. coli* have been developed to restore porin expression and enhance antibiotic uptake. OmpF facilitates the entry of antibiotics such as β-lactams and quinolones, but its expression is repressed by MicF under stress conditions, thereby limiting membrane permeability and promoting resistance. Recent advances in synthetic biology, such as cell-free transcription–translation systems, have identified short ASOs capable of interfering with MicF binding to *ompF* mRNA. Although an initial 12-nucleotide ASO showed limited success in improving antibiotic susceptibility, a dual-ASO strategy targeting the MicF-*ompF* interaction region comprehensively enhanced bacterial sensitivity to cephalothin and nalidixic acid, demonstrating the therapeutic potential of ASOs (Tsai et al., 2023).

ASOs can also target quorum sensing (QS)-associated sRNAs, which play crucial roles in regulating bacterial virulence, biofilm formation, and antibiotic resistance. For instance, in *V. cholerae*, **Qrr** sRNAs repress the master regulator HapR at low cell densities, promoting virulence factor production and host colonization. ASOs designed to sequester Qrr sRNAs prevent their interaction with *hapR* mRNA, effectively locking *V. cholerae* in a high-cell-density state which suppresses virulence and limits infection. This highlights the potential of ASOs to modulate complex regulatory circuits, such as QS, which are key contributors to bacterial survival, pathogenesis, and resistance mechanisms (Feng et al., 2015; Henderson et al., 2021b).

In addition to sRNAs, targeting their associated protein interaction partners is another promising strategy. The Hfq-sRNA system has emerged as an attractive therapeutic target because it is essential for stabilizing sRNA interactions with target mRNAs in Gram-negative pathogens. Disruption of this system impairs the bacterial virulence and adaptation to stress. A notable example is the discovery of RI20, a cyclic peptide identified using split-intein circular ligation of peptides and proteins screening technology which effectively blocks Hfq-sRNA interactions (El-Mowafi et al., 2014). By targeting sRNAs, such as RybB and MicF, RI20 disrupts key regulatory pathways involved in membrane protein expression and antibiotic resistance. Although still in its early stages, this approach complements ASO-based strategies by offering a broader mechanism for disrupting sRNA-mediated regulatory networks, particularly in multidrug-resistant Gram-negative bacteria.

CsrA offers another therapeutic target owing to its critical role in regulating mRNA stability and translation initiation. Compounds that inhibit CsrA-RNA interactions, such as the myxobacterial metabolite MM14, indirectly modulate sRNA-regulated pathways, including those governing virulence factor expression and exopolysaccharide synthesis. This strategy provides an additional layer of control over bacterial adaptation and resistance mechanisms and can potentially enhance the efficacy of existing antibiotics when used in combination therapies (Maurer et al., 2016).

Finally, synthetic sRNAs have emerged as promising therapeutic agents engineered to specifically downregulate essential bacterial genes or virulence factors. Antimicrobials offer a targeted approach which preserves the host microbiome. Nonetheless, the effective delivery of therapeutic sRNAs remains a significant challenge. To address this, various strategies, including cell-penetrating peptides, nanoparticles (e.g., gold and lipid nanoparticles), and biological carriers such as outer membrane vesicles (OMVs), have been explored (Ajam-Hosseini et al., 2023; Paunovska et al., 2022). In particular, non-viral delivery platforms hold significant promise because of their reduced immunogenicity and adaptability to modifications. Successful delivery systems must ensure efficient intracellular uptake, sustained release, and targeted delivery while avoiding degradation and triggering adverse immune responses (Paunovska et al., 2022). Preclinical studies have demonstrated the potential of these delivery technologies; however, translating these findings into clinical practice requires overcoming several challenges. These include improving the delivery efficiency, minimizing off-target effects, scaling up production for clinical use, and meeting stringent regulatory standards. Addressing these hurdles is essential to unlock the full therapeutic potential of sRNAs and establish them as viable tools to combat MDR pathogens and other critical healthcare challenges.

## Conclusion

Progressive increase in the prevalence MDR pathogens poses a significant threat to global public health, emphasizing the need for urgently developing novel antimicrobial strategies. With their ability to regulate gene expression post-transcriptionally, sRNAs have emerged as crucial players in regulating antibiotic resistance mechanisms. By modulating essential processes, such as drug uptake, efflux pump activity, envelope modification, biofilm formation, and DNA mutagenesis, sRNAs contribute to bacterial survival under antimicrobial pressure, highlighting their potential as therapeutic targets.

The growing interest in sRNA research, which parallels the progress of microRNA (miRNA)-based therapies in eukaryotic systems, highlights the potential of sgRNA-based therapeutics for combating antibiotic resistance. These therapies aim to disrupt bacterial resistance pathways by either inhibiting resistance-associated sRNAs or mimicking sRNA activity to restore the antibiotic susceptibility of resistant strains. Such strategies could complement existing antibiotics and enhance their efficacy while reducing the emergence of resistance. However, despite these promising developments, several significant challenges still exist. Efficient delivery systems must ensure stability, specificity for bacterial targets, and scalability for clinical applications. Recent advances in the use of nanoparticles and OMVs as delivery platforms have provided hope for overcoming these hurdles; however, further innovation is required.

Bacterial sRNAs represent a promising yet underexplored frontier in the fight against MDR pathogens. To fully realize their therapeutic potential, interdisciplinary efforts that integrate molecular biology, bioinformatics, and clinical research are essential. By addressing the current challenges and leveraging recent advancements, sRNA-based therapeutics could play a pivotal role in overcoming the global antibiotic resistance crisis.

## Figures and Tables

**Table 1.** Summary of trans-acting sRNAs contributing to antimicrobial resistance or susceptiblity in this study

sRNAs	Target	Regulation of target	Mechanism of action	Bacterial species	Antibiotic(s)	References
DsrA	*mdtEF*	Activation	Multidrug Efflux	*E. coli*	oxacillin, cloxacillin, erythromycin, novobiocin	Nishino et al. (2011)
GlmY/GlmZ	*glmS*	Activation	Envelope modification	*E. coli, Salmonella*	Nva-FMDP, Bacilysin	Khan et al. (2016)
MTS1338	*cydC*	Activation	Multidrug Efflux	*Mycobacterium tuberculosis*	rifampicin	Singh and Dutta (2024)
PhaS	*phoP*	Activation	Envelope modification	carbapenem-resistant *Klebsiella pneumoniae*	polymyxin B	Zhao et al. (2024)
RNA00203	*pgaB*	Activation	Biofilm formation	*Acinetobacter baumannii*	imipenem, ciprofloxacin	Shenkutie et al. (2023)
RyhB	*cirA*	Activation	Drug uptake	*E. coli*	colicin Ia	Salvail, et al. (2013)
Sr006	*pagL*	Activation	Envelope modification	*Pseudomonas aeruginosa*	polymyxin	Zhang, et al. (2017)
sRNA1039	*cfa*	Activation	Drug uptake	*Shigella sonnei *	ampicillin, gentamicin, cefuroxime	Du et al. (2024)
sRNA1600	*tomB*	Activation	Formation of persister cells	*Shigella sonnei *	cefuroxime	Du et al. (2024)
GcvB	*cycA*	Repression	Drug uptake	*E. coli*	D-cycloserine	Pulvermacher et al. (2009)
MgrR	*eptB*	Repression	Envelope modification	*E. coli*	polymyxin B	Moon and Gottesman (2009)
MicA (SraD)	*phoP/Q*	Repression	Envelope modification	*E. coli*	antimicrobial peptides	Coornaert et al. (2010)
MicC	*ompC/N*	Repression	Drug uptake	*E. coli*	β-lactam	Chen et al. (2004), Dam et al. (2017)
MicF	*ompF*	Repression	Drug uptake	*E. coli, Salmonella*	cephalosporins, norfloxacin	Kim et al. (2015)
NrrF	*mtrF*	Repression	Multidrug Efflux	*Neisseria gonorrhoeae*	sulfonamides	Su et al. (2015)
RsaOI	*hpr, atl*	Repression	Envelope modification	*Staphylococcus aureus*	vancomycin	Wu et al. (2024)
RyeB (SdsR)	*tolC*	Repression	Multidrug Efflux	*E. coli, Salmonella*	novobiocin, levofloxaci	Kim, et al. (2015), Parker and Gottesman (2016)
RyeB (SdsR)	*mutS*	Repression	DNA mutagenesis	*E. coli*	ampicillin	Gutierrez et al. (2013)
SprX (RsaOR)	*spoVG*	Repression	Multiple pathways	*Staphylococcus aureus*	glycopeptides	Eyraud et al. (2014)
Sr0161, ErsA	*oprD*	Repression	Drug uptake	*Pseudomonas aeruginosa*	carbapenem (meropenem)	Zhang et al. (2017)
SroC	MgrR	Repression	Envelope modification	*Salmonella*	polymyxin B	Acuna et al. (2016)
